# Should kidney volume be used as an indicator of surgical occasion for patients with autosomal dominant polycystic kidney disease?

**DOI:** 10.1097/MD.0000000000011445

**Published:** 2018-07-06

**Authors:** Jiang Yu, Bin Li, Yu-zhu Xiang, Tai-guo Qi, Xun-bo Jin, Hui Xiong

**Affiliations:** a Minimally Invasive Urology Center, Shandong Provincial Hospital Affiliated to Shandong University; b Medical School, Shandong University, Jinan, China.

**Keywords:** autosomal dominant polycystic kidney disease, decortication, laparoscopy, renal volume

## Abstract

To investigate the best surgical occasion of laparoscopic cyst decortications (LCDs) in patients with autosomal dominant polycystic kidney disease (ADPKD), in accordance with the renal volume (RV). We retrospectively analyzed 135 (65 male and 70 female) patients with ADPKD who underwent LCD between June 2011 and October 2015. Patients were divided into 4 groups according to the volume of the operated kidney measured from computed tomography scans: group A (28 patients, RV < 500 mL), group B (63 patients, RV = 500–1000 mL), group C (30 patients, RV = 1000–1500 mL), and group D (14 patients, RV > 1500 mL). We studied postoperative indicators at least 1-year follow-up. For each RV group, therapeutic responses of LCD in these patients with ADPKD were assessed by improvement of clinical parameters and manifestations. A significant glomerular filtration rate (GFR) improvement was found in RV group B (31.8 ± 11.1 mL/min; final GFR 36.9 ± 12.7 mL/min; *P* < 0.01), and RV group C (21.1 ± 8.7 mL/min; final GFR 27.4 ± 9.2 mL/min; *P* < 0.01). RV group C had much higher GFR improvements than did RV group B (*P* < 0.01). In addition, refractory pain in patients of RV groups B, C, and D was much relieved by LCD treatment. Compared with other RV groups, blood pressures in patients with ADPKD of RV group D were also improved (*P* < 0.01). Our study indicates that RV could be used to evaluate LCD clinical outcomes in patients with ADPKD. The results of LCD for patients with ADPKD with RV between 500 and 1500 mL were encouraging, especially with regards to renal function improvement and pain relief. Therefore, RV may become a useful marker to predict the timing of LCD surgery in patients with ADPKD.

## 1 Introduction

Autosomal dominant polycystic kidney disease (ADPKD) is the most common hereditary renal disease affecting all ethnic groups worldwide with an incidence of 1:500 to 1000. Renal cysts grow exponentially in ADPKD.^[1,2]^ Laparoscopic cyst decortication (LCD) has been widely applied in patients with ADPKD, which reduces renal mass, decreases hydrostatic pressure in cysts, diminishes pain, is beneficial for blood pressure (BP) management, and postpones disease progression into end-stage renal disease (ESRD).^[3]^ However, controversy remains over the timing of LCD surgery in these patients with ADPKD. Some studies indicated that the LCD could be carried out when the single cyst is larger than 4 cm in diameter. LCD could also be considered when hematuria flank pain occurred.^[4]^ Furthermore, a study suggested that ADPKD should be treated with LCD as early as possible^[5]^ because patients with giant kidney volume would not benefit from LCD treatment.^[6]^ Although there is still no widely accepted indication for LCD in ADPKD treatment, several studies have shown that renal volume (RV) is significantly associated with the clinical outcomes of patients with ADPKD.^[2,6–10]^ However, to our knowledge, no reports have demonstrated whether RV could be used to evaluate LCD clinical outcome in these patients. In this study, we retrospectively enrolled patients with ADPKD who have received LCD treatment in our hospital since 2007. By analyzing preoperative and postoperative RV, hypertension, flank pain, and renal functions (glomerular filtration rate, GFR), we determined whether RV is a potential marker for LCD treatment in patients with ADPKD and which RV stage of ADPKD is appropriate to use LCD treatment.

## 2 Research design and study conduct

Between June 2013 and January 2016, 163 patients with ADPKD were admitted to our hospital and 28 patients were excluded (23 patients chose conservative therapy and 5 patients lacked 12 months follow-up data after surgery). In total, 135 cases of enrolled patients (65 male and 70 female) underwent unilateral LCD. Intraoperative observed indicators, including operative duration (OD), bleeding volume (BV), blood transfusion rate (BTR), days of postoperative hospitalization (DPH), and postoperative complications were recorded and compared. All these patients were followed-up postoperatively for at least 1 year and clinical data were recollected at 1 year after surgery, including GFR, BP, and pain levels. Patients were divided into 4 groups according to the volume of the operated kidney measured from computed tomography (CT) scans: group A (28 patients, RV < 500 mL), group B (63 patients, RV = 500–1000 mL), group C (30 patients, RV = 1000–1500 mL), and group D (14 patients, RV > 1500 mL). Table 1 summarizes patients’ characteristics.

**Table 1:**
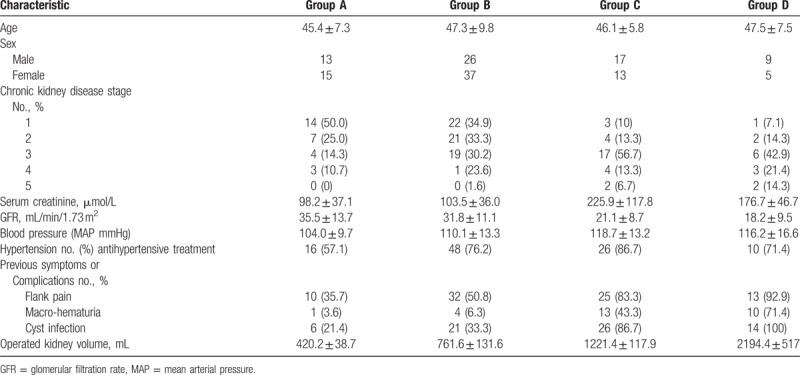
Characteristics of ADPKD patients of different groups.

## 3 Measurement and definitions

### 3.1 Criterion for LCD surgery

Patients diagnosed with ADPKD with a single cyst larger than 4 cm in diameter and with hypertension, hematuria, cyst infection, or refractory pain will be recommended to receive a single LCD surgery. Patients with severe kidney pain or unilateral enlarged RV were eligible for surgery.

### 3.2 Surgical procedure

All 135 cases underwent laparoscopic unilateral decortication of polycystic kidney by intraperitoneal route. Patients lay on their healthy side at a 20° incline and general anesthesia was achieved via tracheal intubation. After pneumoperitoneum was established below the connection of the costal margin and the midclavicular line, we used a 10-mm puncture needle to successfully puncture parallel to the umbilical parasternal line and then below the costal margin anterior axillary line to establish pneumoperitoneum with a 5-mm puncture. A longitudinal incision was made on retroperitoneum along with lateral sulci with ultrasonic scalpel to fully open renal fascia and free the kidney. Cysts with a large volume and high tension that were visible on the kidney surface were prioritized for excision via cyst wall resection and other visible cysts were subsequently excised. Laparoscopic ultrasound imaging was used to guide drainage of the grossly undetectable cysts.

### 3.3 Intraoperative observed indicators

Indicators included OD, BV, BTR, DPH (Table 2), and postoperative complications (such as urine leakage, intestinal adhesion and obstruction, retroperitoneal hematoma; Table 3).

**Table 2:**
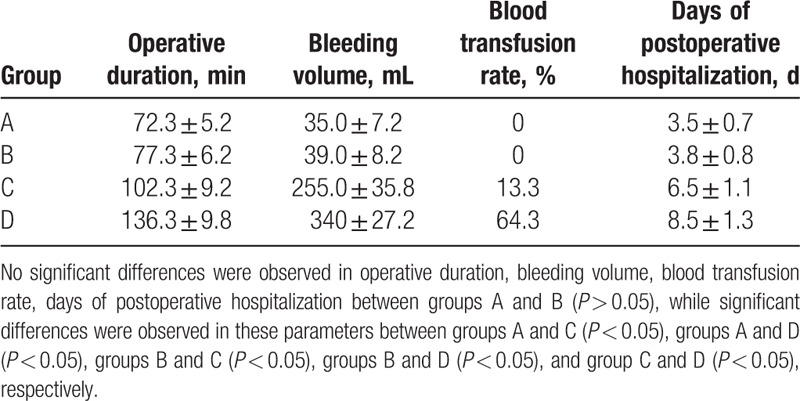
The comparison of operative data between patients in 4 groups.

**Table 3:**
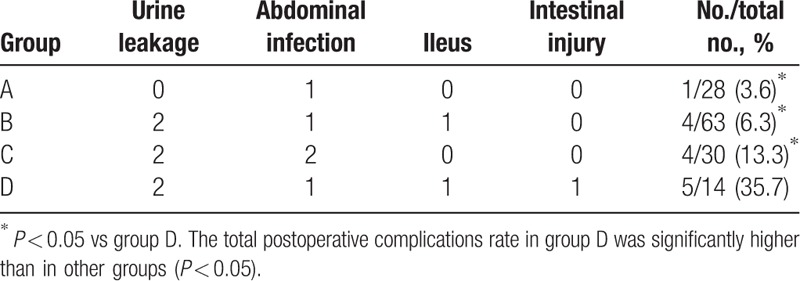
The comparison of postoperative complications in 4 groups.

### 3.4 Renal function analyses

Renal function was evaluated by GFR (renal dynamic Tc99 scintigraphy). All data were collected preoperatively, postoperatively at the 12th month and each follow-up visit (Table 4).

**Table 4:**
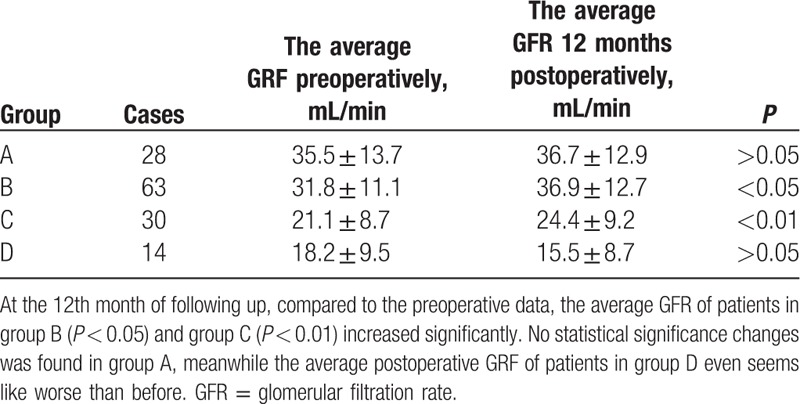
The comparison of GFR preoperatively and postoperatively in 4 groups.

### 3.5 Blood pressure control

The effect of LCD on BP control was evaluated by use of the antihypertensive therapeutic index (ATI), measured with the formula ([dose of BP medication 1/maximum permissible dose 1] + [dose of BP medication 2/maximum permissible dose 2] + etc) × 10.^[10]^ The ATI was calculated preoperatively and at each follow-up (Table 5).

**Table 5:**
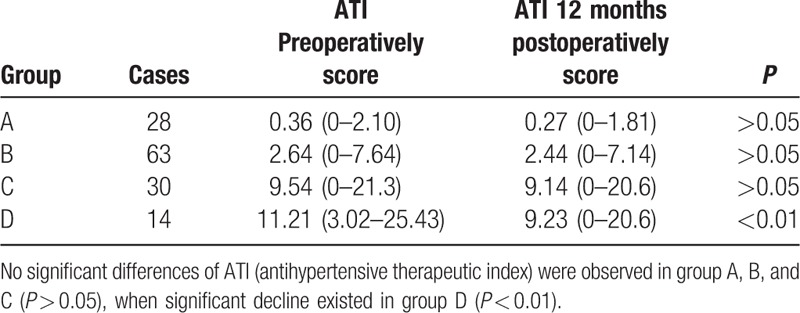
The comparison of hypertension preoperatively and postoperatively in 4 groups.

### 3.6 Pain control

Pain assessment was based on a telephone or interview questionnaire. Pain relief was assessed using a pain analog scale; relative pain relief (RPR) equaled ([preoperative pain score] − [postoperative pain score])/(preoperative pain score) (Table 6).

**Table 6:**
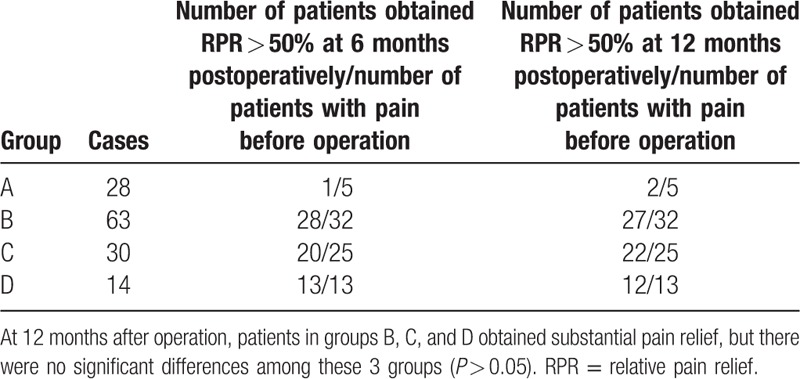
The pain control effect in 4 groups.

### 3.7 Renal volume calculation

Length, width, and thickness of both kidneys of the patients with CT before surgery were measured by 2 surgeons independently and the mean value was calculated. The improved elliptic volume formula was used to calculate RV. RV (μL) = (4/3)π × (width/4 + thickness/4)^2^ × (length/2).^[7]^

### 3.8 Statistical analyses

SPSS 17.0 software, IBM, Armonk, NY was used to perform the Student *t* test, Fisher exact test, and χ^2^ test when comparing differences in means and proportions, respectively. The χ^2^ test or Fisher exact test was used for categorical variables and the Student *t* test was used for continuous variables. All tests were 2-tailed and were defined as significant for *P* < 0.05.

## 4 Results

### 4.1 Operative data

A total of 135 procedures in 65 men and 70 women were performed. There were no perioperative deaths. Detailed intraoperative data were showed in Table 2. The ODs in groups A to D were 58 to 93 minutes (72.3 ± 5.2 minutes), 63 to 102 minutes (77.3 ± 6.2 minutes), 93 to 135 minutes (102.3 ± 9.2 minutes), and 102 to 175 minutes (136.3 ± 9.8 minutes), respectively. BV in groups A to D were 15 to 65 mL (35.0 ± 7.2 mL), 25 to 75 mL (39.0 ± 8.2 mL), 150 to 620 mL (255.0 ± 35.8 mL), and 230 to 850 mL (340 ± 27.2 mL), respectively. No patient required a blood transfusion in groups A and B, meanwhile 4 (13.3%, 4/30) patients in group C and 9 (64.3%, 9/14) patients in group D received a blood transfusion. The DPH in groups A to D were 3 to 5 days (3.5 ± 0.7 days), 3 to 6 days (3.8 ± 0.8 days), 5 to 9 days (6.5 ± 1.1 days), and 7 to 11 days (8.5 ± 1.3 days), respectively. No significant differences were observed in OD, BV, BTR, and DPH between groups A and B (*P* > 0.05). Significant differences were observed in OD, BV, DPH between groups A and C, groups A and D, groups B and C, groups B and D, and groups C and D (*P* < 0.05).

### 4.2 Postoperative complications

Postoperative complications mainly included urine leakage (B: 2/63, C: 2/30, D: 2/14), abdominal infection (A: 1/28, B: 1/63, C: 2/30, D: 1/14), ileus (B: 1/63, D: 1/14), and intestinal injury (D: 1/14 Table 3). The total postoperative complications rate in group D was significantly higher than in other groups (*P* < 0.05).

### 4.3 Renal function outcomes

**Figure 1:**
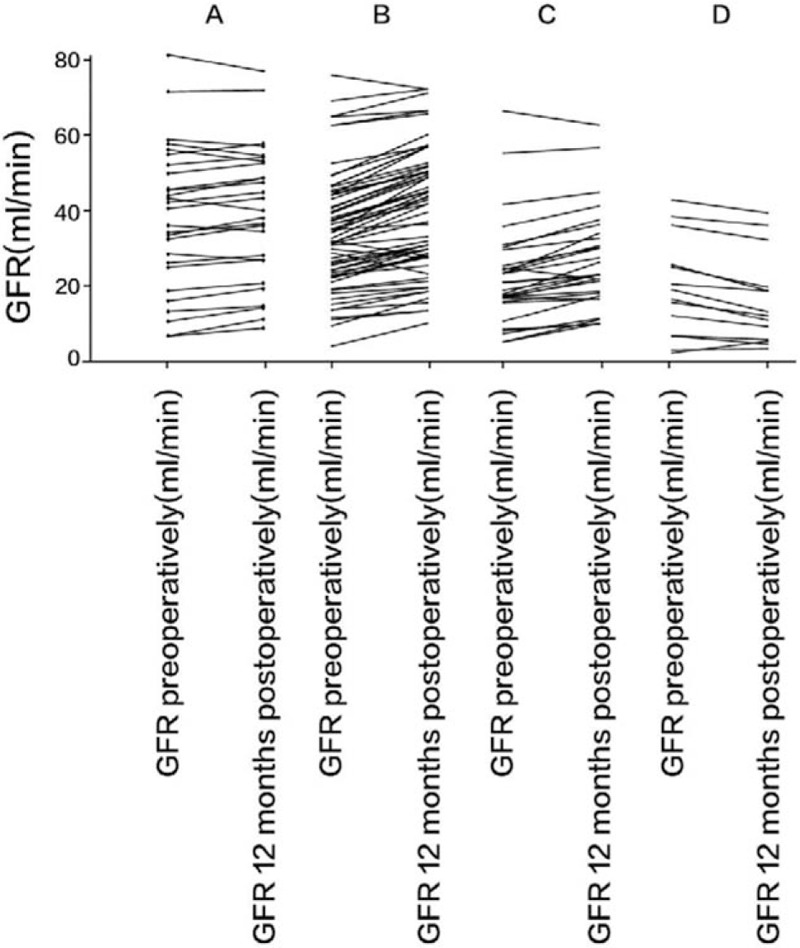
Trends in glomerular filtration rate (GFR) in each patient in groups A, B, C, and D 12 months postoperatively. Postlaparoscopic cyst decortication trends in GFR of each patient in 4 groups, respectively. The ordinate scale represents the GFR value of each patient before and 12 months after surgery, and the line segments intuitively show the change of GFR of each patient.

At the 12th month of follow-up, compared with preoperative data, the GFR of patients in groups B and C increased significantly (*P* < 0.05; Table 4). Moreover, the GFR increase in group C was statistically greater than that of group B (*P* < 0.01). Meanwhile, there was no statistical significance in GFR changes was found in groups A and D (*P* > 0.05). Figure 1 illustrates post-LCD trends in GFR of each patient in groups A to D who underwent this procedure.

### 4.4 Hypertension outcomes

No significant differences of hypertension were observed in groups A to C preoperatively and postoperatively (*P* > 0.05). Group D was significantly different from groups A to C (*P* < 0.01; Table 5).

### 4.5 Pain control outcomes

Following the alleviation of flank pain, significantly increased bloating was seen in groups B, C, and D compared with presurgery (*P* < 0.01), but there were no significant differences among these 3 groups (*P* > 0.05; Table 6).

## 5 Discussion

ADPKD is the most common hereditary renal disease that could progress into ESRD, and there is no radical treatment. Of all patients with ADPKD, at least half will progress to ESRD by age 60 years.^[11,12]^ In 1911, Rovsing^[13]^ initially reported 3 patients in which cysts were treated with pain relief and renal functional improvement was observed. However, in *New England Journal of Medicine* in 1957, Bricker and Patton^[14]^ reported 2 patients with mild renal insufficiency preoperatively and both patients had worsening renal function after cyst decortication. Therefore, research into cyst decortication for ADPKD was abandoned for nearly 20 years. Until, in the 1980s, Ye et al^[15]^ reported open surgical cyst decortication for ADPKD again. They analyzed 96 patients with ADPKD; 91% and 77% patients acquired pain relief in 6 months and 5 years after cyst decortication, respectively. Most importantly, no deterioration in renal function was observed postoperatively. Subsequently, research interest in surgical cyst decortication for ADPKD increased. In 1996, Elashry et al^[16]^ initially reported 5 LCD procedures in ADPKD. In 2012, Haseebuddin et al^[3]^ examined the long-term impact of LCD on renal function, hypertension, and pain control in patients with ADPKD, at a mean follow-up of 10.9 years, and noted that a cautious approach with LCD should be taken in patients with poor preoperative renal function. At this time, there were still some controversies in LCD for ADPKD,^[17]^ for example, regarding the most appropriate indicator for the surgery of ADPKD,severe back pain, uncontrollable hypertension, hematuria, recurrent infections, or CKD stage based on the National Kidney Foundation staging system?

The RV could be a marker of increasing cyst size. In recent years, compared with clinical symptoms or imaging results of B-scan ultrasonography and other techniques, some studies reported that RV is the most sensitive indicator of polycystic kidney disease progression,^[18,19]^ especially in the early progression prediction.^[20–22]^ In addition, in 2002, Fick-Brosnahan et al^[7]^ conducted the first sequential quantitative study of total kidney volumes (TKVs) in adults with ADPKD. They followed 229 patients over a mean interval of 7.8 years and their results indicated that there is a significant correlation between increased total RV and declined GFR.^[7]^ In 2006, the Consortium for Radiologic Imaging Studies of Polycystic Kidney Disease Group found that kidney enlargement resulting from the expansion of cysts in patients with ADPKD is continuous and quantifiable and is associated with the decline of renal function. Higher rates of kidney enlargement are associated with a more rapid decrease in renal function.^[2]^ A subsequent study demonstrated a significant relationship between the change in renal function and the change in RV in a Japanese patient with ADPKD without renal insufficiency. This study concluded that volume measurements can be used as useful markers for disease progression in Japanese patients with ADPKD,^[8]^ and other scholars found that baseline height-adjusted TKV (htTKV) > 600 cm^3^/m predicted the risk of developing renal insufficiency in patients with ADPKD at high risk for renal disease progression within 8 years of follow-up, thus qualifying htTKV as a prognostic biomarker in ADPKD.^[9]^

Therefore, we speculated that RV could be an operative indication of decortication for ADPKD. To our knowledge, this is the first study investigating RV as an indicator for LCD surgery of ADPKD. When the RV was <500 mL, the pressure on the renal parenchyma was maintained in a low level, so renal function was not significantly impaired with a normal GFR. Therefore, LCD procedure could not improve GFR level. In this study, patients in group A did not benefit from LCD. No significant differences of ATI and RPR were observed in group A preoperatively and postoperatively. When the RV increased to 500 to 1500 mL, the kidney started to decompensate with a rapid decline GFR. At the same time, the morbidity of complications, such as hypertension, abdominal compression, pain, and macroscopic hematuria, significantly increased along with increasing RV. For patients in this period of time, a procedure could be performed to remove the cysts, which would relieve pressure on renal parenchyma, improve renal blood supply, maximize the recovery of renal function, postpone disease progression, and control the clinical symptoms. In this study, the average GFR level of groups B and C increased at 1 year after LCD, similar results were reported by Fryczkowski et al,^[23]^ but no improvement in mean ATI was noted. Due to serve kidney injury in bilateral procedure of renal cyst decortications, the procedures of each side could be performed separately, but this would delay the treatment and lead to deterioration. If the RV was >1500 mL, the kidney would progress into a state of decompensation ability with about a 10% GFR decrease of normal levels, and more serious complications would occur. For patients in this period, the LCD could not improve the renal function significantly, although it might alleviate some symptoms, including abdominal compression, pain, and other complications. In our experience, the renal function of patients with ADPKD would not be improved by LCD when the RV is >1500 mL; moreover, the transfusion rate and postoperative complications rate significantly increased in those patients.

All the patients in our study only received unilateral surgery, so the benefit of BP control was limited. Although we assumed that a bilateral procedure would improve BP control more significantly, which warrants further study.

In conclusion, we believe that it essential that a sensitive marker is found to evaluate the progression of polycystic kidney disease in order to guide clinical treatment and surgical indication. Previous studies have shown that both the conventionally adopted serum creatinine concentration and GFR were not as sensitive as expected at predicting polycystic kidney disease progression, especially in the early stages. Furthermore, GFR measurement is difficult to popularize due to instruments and medicament. Our results indicate that RV is a more sensitive indicator to reflect polycystic kidney disease progression. Advantages of RV test include ease, low cost, and noninvasive. Therefore, RV could play a role in the determination of surgical indication and provide some useful information about the procedure outcome.

## 6 Conclusion

As an LCD operative indicator of polycystic kidney, RV was efficient and feasible. Besides, other factors should be considered as the operative indication, which could involve the outcome of surgery. The results of LCD for patients with ADPKD with a RV between 500 and 1500 mL were encouraging, especially in renal function improvement and pain relief. As a retrospective cohort study, there were still some limitations to our study, so further study with randomized prospective studies is warranted, with more samples and long-term follow-up to confirm the conclusion.

## Acknowledgments

The authors thank Dr Xu Li-na and Dr Yuan Hang for assistance in data collection; Dr Xia Ting from Zoucheng Renmin Hospital for providing the conception of the study. The department of Nuclear Medicine of our hospital to offer renal dynamic Tc99 scintigraphy service. This work is supported by Shandong Medical and Health Science and Technology Development Program (2016WSB01033) and Shandong Key Research and Development Plan (2018GSF118189).

## Author contributions

Xiang Yu-zhu (XY), Qing Tai-guo (QT) and Jin Xun-bo (JX) participated in the patient selection and surgical preparation. Li Bin participated in the data collection and following up management. Yu Jiang carried out the data interpretation, analyses and manuscript writing. Xiong Hui participated in the design of the study and surgery of all patients. All authors read and approved the final manuscript.

**Data curation:** Bin Li, Zhu yu Xiang.

**Formal analysis:** Bo xun Jin.

**Software:** Guo tai Qi.

**Writing – original draft:** Jiang Yu.

**Writing – review & editing:** Hui Xiong.

## References

[1] Torres VE Harris PC Pirson Y Autosomal dominant polycystic kidney disease. Lancet 2007;369:1287–301. 17434405 10.1016/S0140-6736(07)60601-1

[2] Grantham JJ Torres VE Chapman AB Volume progression in polycystic kidney disease. N Engl J Med 2006;354:2122–30. 16707749 10.1056/NEJMoa054341

[3] Haseebuddin M Tanagho YS Millar M Long-term impact of laparoscopic cyst decortication on renal function, hypertension and pain control in patients with autosomal dominant polycystic kidney disease. J Urol 2012;188:1239–44. 22902029 10.1016/j.juro.2012.06.026

[4] Meijer E Rook M Tent H Early renal abnormalities in autosomal dominant polycystic kidney disease. Clin J Am Soc Nephrol 2010;5:1091–8. 20413443 10.2215/CJN.00360110 PMC2879311

[5] Torres VE Treatment strategies and clinical trial design in ADPKD. Adv Chronic Kidney Dis 2010;17:190–204. 20219622 10.1053/j.ackd.2010.01.006 PMC4127876

[6] Alam A Perrone RD Management of ESRD in patients with autosomal dominant polycystic kidney disease. Adv Chronic Kidney Dis 2010;17:164–72. 20219619 10.1053/j.ackd.2009.12.006

[7] Fick-Brosnahan GM Belz MM McFann KK Relationship between renal volume growth and renal function in autosomal dominant polycystic kidney disease: a longitudinal study. Am Kidney Dis 2002;39:1127–34. 10.1053/ajkd.2002.33379 12046022

[8] Tokiwa S Muto S China T The relationship between renal volume and renal function in autosomal dominant polycystic kidney disease. Clin Exp Nephrol 2011;15:539–45. 21431900 10.1007/s10157-011-0428-y

[9] Chapman AB Bost JE Torres VE Kidney volume and functional outcomes in autosomal dominant polycystic kidney disease. Clin J Am Soc Nephrol 2012;7:479–86. 22344503 10.2215/CJN.09500911 PMC3302672

[10] Woon C Bielinski-Bradbury A O’Reilly K A systematic review of the predictors of disease progression in patients with autosomal dominant polycystic kidney disease. BMC Nephrol 2015;16:1–6. 10.1186/s12882-015-0114-5 PMC4536696 26275819

[11] Parfrey PS Bear JC Morgan J The diagnosis and prognosis of autosomal dominant polycystic kidney disease. N Engl J Med 1990;323:1085–90. 2215575 10.1056/NEJM199010183231601

[12] Gabow PA Johnson AM Kaehny WD Factors affecting the progression of renal disease in autosomal-dominant polycystic kidney disease. Kidney Int 1992;41:1311–9. 1614046 10.1038/ki.1992.195

[13] Rovsing T Treatment of multilocular renal cyst with multiple punctures. Hospitalstid 1911;4:105.

[14] Bricker NS Patton JF Renal function studies in polycystic disease of the kidneys. N Engl J Med 1957;256:212. 13400236 10.1056/NEJM195701312560504

[15] Ye M An SY Jiang HM Clinical analysis of 141 cases of adult polycystic kidney disease. Zhonghua Wai Ke Za Zhi 1986;24:73. 3743269

[16] Elashry OM Nakada SY Wolf JS Jr Laparoscopy for adult polycystic kidney disease: a promising alternative. Am J Kidney Dis 1996;27:224–33. 8659498 10.1016/s0272-6386(96)90545-4

[17] Millar M Tanagho YS Haseebuddin M Surgical cyst decortication in autosomal dominant polycystic kidney disease. J Endourol 2013;27:528–34. 23157176 10.1089/end.2012.0529 PMC3643310

[18] Sans Atxer L Roca-Cusachs A Torra R Relationship between renal size and blood pressure profile in patients with autosomal dominant polycystic kidney disease without renal failure. Nefrologia 2010;30:567–72. 20882096 10.3265/Nefrologia.pre2010.May.10418

[19] Cadnapaphornchai MA Masoumi A Strain JD Magnetic resonance imaging of kidney and cyst volume in children with ADPKD. Clin J Am Soc Nephrol 2011;6:369–76. 21115621 10.2215/CJN.03780410 PMC3052228

[20] Ekser B Rigotti P Images in clinical medicine. Autosomal dominant polycystic kidney disease. N Engl Med 2010;363:71. 10.1056/NEJMicm0905399 20592299

[21] Mignani R Corsi C De Marco M Assessment of kidney volume in polycystic kidney disease using magnetic resonance imaging without contrast medium. Am Nephrol 2011;33:176–84. 10.1159/000324039 21311183

[22] Lee CC Fang CY Huang CC Computed tomography angiographic demonstration of an unexpected left main coronary artery dissection in a patient with polycystic kidney disease. Thorac Imaging 2011;26:W4–6. 10.1097/RTI.0b013e3181dc2a53 20634758

[23] Fryczkowski M Huk J Sitko-Saucha A Place of laparoscopic cysts decortication (LCD) in the treatment of autosomal dominant polycystic kidney disease (ADPKD). Prog Urol 2007;17:1324–7. 18271415 10.1016/s1166-7087(07)78570-6

